# Does insufficient sleep affect how you learn from reward or punishment? Reinforcement learning after 2 nights of sleep restriction

**DOI:** 10.1111/jsr.13236

**Published:** 2020-11-20

**Authors:** Andreas Gerhardsson, Danja K. Porada, Johan N. Lundström, John Axelsson, Johanna Schwarz

**Affiliations:** ^1^ Department of Psychology Stockholm University Stockholm Sweden; ^2^ Department of Psychology Stress Research Institute Stockholm University Stockholm Sweden; ^3^ Department of Clinical Neuroscience Karolinska Institute Stockholm Sweden; ^4^ Monell Chemical Senses Center Philadelphia PA USA; ^5^ Department of Psychology University of Pennsylvania Philadelphia PA USA; ^6^ Stockholm University Brain Imaging Centre Stockholm University Stockholm Sweden

**Keywords:** carrot or stick, feedback‐based learning, lack of sleep, reward or punishment, sleep deprivation, valanced feedback

## Abstract

To learn from feedback (trial and error) is essential for all species. Insufficient sleep has been found to reduce the sensitivity to feedback as well as increase reward sensitivity. To determine whether insufficient sleep alters learning from positive and negative feedback, healthy participants (*n* = 32, mean age 29.0 years, 18 women) were tested once after normal sleep (8 hr time in bed for 2 nights) and once after 2 nights of sleep restriction (4 hr/night) on a probabilistic selection task where learning behaviour was evaluated in three ways: as generalised learning, short‐term win–stay/lose–shift learning strategies, and trial‐by‐trial learning rate. Sleep restriction did not alter the sensitivity to either positive or negative feedback on generalised learning. Also, short‐term win–stay/lose–shift strategies were not affected by sleep restriction. Similarly, results from computational models that assess the trial‐by‐trial update of stimuli value demonstrated no difference between sleep conditions after the first block. However, a slower learning rate from negative feedback when evaluating all learning blocks was found after sleep restriction. Despite a marked increase in sleepiness and slowed learning rate for negative feedback, sleep restriction did not appear to alter strategies and generalisation of learning from positive or negative feedback.

## 1 INTRODUCTION

Reinforcement learning is how we learn from positive (reward) or negative (punishment) feedback and adapt behaviour to maximise reward (Sutton & Barto, 2018). Stemming from early animal behavioural studies, including Ivan Pavlov’s salivating dogs (Todes, 1997) and Thorndike’s law of effect (Thorndike, 1911), reinforcement learning is now being applied to machine learning and neural signalling (Sutton & Barto, 2018). Sleep loss has previously been found to affect the response to feedback (Liu & Zhou, 2016; Whitney et al., 2015). However, whether sleep loss affects the incentives to learn from positive or negative feedback has not yet been explored.

The detrimental effects on a range of cognitive functions, including attention and working memory, are well documented for total sleep deprivation (Lim & Dinges, 2010; Pilcher & Huffcutt, 1996), but also for shorter periods of sleep restriction (van Dongen et al., 2003; Lowe et al., 2017). Attentional degradation after sleep loss may in turn cause deficits in item and associative recognition memory (Ratcliff & Van Dongen, 2018). Sleep deprivation increases reward‐seeking tendencies (Venkatraman et al., 2011) and causes an over‐activation in the reward‐related neural circuitries in response to gamble wins (Mullin et al., 2013; Venkatraman et al., 2007), positive images (Gujar et al., 2011), and food desirability (Greer et al., 2013; St‐Onge et al., 2012), compared to normal night sleep. These reward‐seeking tendencies may also be related to findings of attenuated risk‐aversion after sleep loss (Killgore, 2015; Maric et al., 2017). Moreover, studies investigating the response to feedback have found reduced event‐related potentials (ERP) amplitudes after 72 hr of sleep deprivation (Liu & Zhou, 2016) and attenuated skin conductance response after 62 hr of sleep deprivation (Whitney et al., 2015), which in the latter study was associated with an ineffective use of feedback, although the authors did not investigate differences in response to feedback.

One of the mechanisms behind a reward‐seeking behaviour after total sleep deprivation can be traced to a reduced availability of D_2_ and D_3_ dopamine receptors (Volkow et al., 2012), which in turn increases the D_1_ receptor activation, making sleep deprived individuals hypersensitive to reward (Krause et al., 2017). Support of a direct link between dopamine availability and reinforcement learning (Garrison et al., 2013) has been found in experimental studies on patients with Parkinson’s disease, where patients *on* dopamine medication prioritised learning from positive feedback while patients *off* medication prioritised avoiding negative feedback (Frank, 2004), or had no specific preference (McCoy et al., 2019). Moreover, reinforcement learning algorithms, such as Q‐learning (Sutton & Barto, 2018), providing a latent measure of the trial‐by‐trial update of the stimuli value have shown that the change in learning rate speed is related to dopamine (McCoy et al., 2019). Indirect evidence on the role of dopamine in reinforcement learning comes from studies on ageing, which leads to increased tendency to avoid negative feedback (Frank & Kong, 2008), and acute stress that can increase the tendency to learn from positive feedback (Lighthall et al., 2013) or reduce the tendency to learn from negative feedback (Petzold et al., 2010).

The main aim of the present study was to determine whether 2 nights of sleep restriction affects the incentives to learn from positive or negative feedback using a probabilistic selection task (Frank, 2004). As there is a scarcity of studies on reward processing after sleep restriction, we rely on findings from total sleep deprivation studies and assume that if sleep restriction, similar to total sleep deprivation, increases the reward incentives (Krause et al., 2017), we would expect that the difference in the proportion of correct responses learned from positive compared to negative feedback would be greater following 2 nights of sleep restriction (~4 hr) than after 2 nights of normal sleep (~8 hr).

We were mainly interested in the generalised reinforcement learning, that is, to what degree participants prioritised to choose the symbol associated with highest positive value (A) against more neutral symbols (C, D, E and F) or to avoid the symbol associated with the highest negative value (B) against more neutral symbols. As sleep loss has been found to affect attention and working memory (Lim & Dinges, 2010; Lowe et al., 2017), we also investigated if the short‐term learning in the initial learning phase was biased towards positive or negative feedback, as shown in other contexts (Lighthall et al., 2013). In addition, we used a computational Q‐learning algorithm to investigate the trial‐by‐trial value update for the learning rate of positive and negative feedback during the learning phase (Frank et al., 2007; McCoy et al., 2019). We measured sleepiness using the Karolinska Sleepiness Scale (KSS; Åkerstedt & Gillberg, 1990) and subjective stress using a rating scale ranging from 1 = very relaxed (“Väldigt avspänd”) to 9 = extremely stressed (“Extremt stressad”) (Schwarz et al., 2018).

## 2 METHODS

### 2.1 Participants

A total of 32 healthy individuals (18 women; mean [*SD*] age 29.0 [7.6] years), recruited from the greater Stockholm area, completed this study in a within‐participant cross‐over fashion. All participants were non‐tobacco users (cigarettes and snus), moderate alcohol and coffee consumers (<3 glasses of alcohol and <6 cups of coffee per day), naïve to the Japanese language, not taking regular medication, not working night shifts, and had normal sleep habits with a habitual sleep requirement of between 7.0 and 9.0 hr. Participants demonstrated normal/corrected‐to‐normal visual and auditory acuity, had no physiological or psychiatric pathology that could affect sleep or any of the measured variables, and had not been travelling across more than two time zones during the previous month. The study was approved by the Regional Ethical Review board in Stockholm, Sweden (DNR: 2010/1506‐31, 2016/64‐32), and conducted in accordance with the Helsinki Declaration. All participants provided written informed consent prior to inclusion and received monetary compensation.

### 2.2 Protocol

Participants were informed about the protocol and performed a training version of the task during an initial screening session. The experimental task was then completed in two test sessions, once after 2 consecutive nights of sleep restriction and once after 2 consecutive nights of normal sleep, in a counterbalanced order with at least a 1‐week wash‐out period of normal sleep between the conditions. Both test sessions took place at the same time of day, with a starting time between 12:30 and 15:00 hours, and began with a calm‐down period of 30 min during which participants completed questionnaires. Subsequently, each participant underwent five to six computerised experimental tasks. The tasks were always presented in the same order, each lasting between 10 and 40 min, with the opportunity to take short breaks in between. After each experimental task, participants completed a short questionnaire about sleepiness and motivation during the just completed task.

In the sleep restriction condition, participants were given a 4‐hr sleep opportunity, the exact timing being self‐chosen between 01:00 hours ± 1 hr and 05:00 hours ± 1 hr. In the normal sleep condition, participants were instructed to make sure to get 7–8 hr time in bed between 23:00 hours ± 1 hr and 07:00 hours ± 1 hr. Both conditions were performed in their homes. Adherence to the protocol was controlled with an actiograph, a wrist‐worn movement sensitive device commonly used to measure sleep–wake activity (Actiwatch, Cambridge Neuro‐Technology Ltd.). Participants were additionally instructed to send a text message to the experimenter shortly before going to sleep and shortly after having woken up. In the morning after each experimental night (2 nights of sleep restriction, 2 nights of normal sleep), participants completed a sleep diary, providing information about sleep times, sleep quality, and the feeling of sleepiness in the morning. Napping, hard physical training, as well as consuming caffeine or alcohol was not permitted 2 days before and the day of the test session. Sleep parameters are presented in Table 1.

**Table 1:** Mean (*SD*) and [range] of actiography sleep parameters calculated from the aggregated means of each participant over the 2 nights of measurement

	Normal sleep	Sleep restriction	BF_10_	BF_01_
Actiography				
Time in bed	07:55 (00:24) [06:58–08:39]	04:09 (00:12) [03:43–04:40]	>30	<1/30
Sleep start	23:46 (00:59) [22:16–03:00]	01:18 (00:44) [23:56–03:45]	>30	<1/30
Sleep end	07:26 (01:03) [05:54–10:40]	05:16 (00:44) [03:50–07:12]	>30	<1/30
Assumed sleep	07:40 (00:27) [06:50–08:59]	03:58 (00:12) [03:27–04:26]	>30	<1/30
Sleep diary				
Well rested (5 = fully, 1 = not at all)	3.6 (0.9) [1.5–5]	1.7 (0.6) [0.5–3]	>30	<1/30
Easy to get up (5 = very easy, 1 = very difficult)	3.7 (0.8) [2–5.0]	2.3 (0.8) [1–4.5]	>30	<1/30

Bayes factors were estimated using Bayesian paired *t* tests with default weakly informative priors (Morey & Rouder, 2015). BF_10_ >30 and BF_01_ <1/30 indicates very strong evidence for a difference.

### 2.3 Probabilistic selection task

**FIGURE 1:**
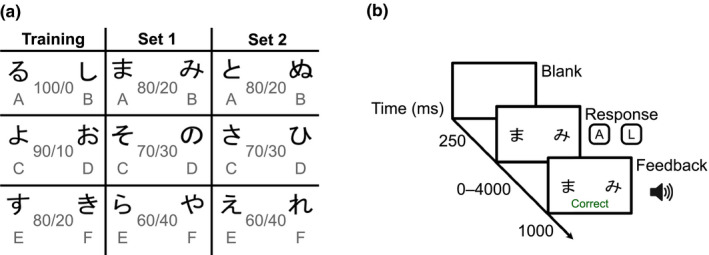
(a) Symbol pairs in the training and the two learning sets together with winning probability within each pair. Learning criteria for Set 1 and Set 2 were ≥65% A choices for A/B, ≥60% C choices for C/D, and ≥40% E choices for E/F after each block. (b) Trial example from learning phase. In the test phase (not depicted) no feedback was given and symbol pairs were scrambled

To test reinforcement learning, we used a probabilistic selection task (Frank, 2004). This task consists of a learning phase and a test phase. In the learning phase participants learn from feedback to choose the most likely winner in a pair of ambiguous symbols. Three pairs (A/B, C/D, E/F, see Figure 1a) of Japanese Hiragana symbols were presented in a pseudorandomised order on the left and right of the centre of the screen (balanced). Within each pair one symbol was more likely to be the winning symbol, which is to render positive feedback (for the pair A/B the ratio was 80/20, for C/D 70/30 and for E/F 60/40). Each trial (Figure 1b) started with a short inter‐trial interval (250 ms) after which the symbols appeared until a response was given or 4,000 ms had passed, in which case the participant was informed that the response was missed. Responses were made by pressing on a keyboard, *A* for the left symbol or *L* for the right symbol and participants were instructed to answer as fast and correctly as possible. Following a response, positive feedback was given as green text stating the Swedish word for “Correct” (Rätt) combined with a sound with ascending pitch, and negative feedback was given as red text stating the Swedish word for “Wrong” (Fel) combined with a sound with a descending pitch. A block consisted of 60 trials where each of the three pairs of symbols was presented 20 times, with balanced lateralisation within the pairs. The learning phase was finished after a block when the participant had reached the learning criteria for all pairs (≥65% A choices for A/B trials, ≥60% C choices for C/D trials, and ≥40% E choices for E/F trials), or reached a maximum of six blocks. In addition, we used a training version of the task at screening, with a different set of characters and more deterministic probabilities, but higher learning criteria. This was done mainly to facilitate the understanding of the task, and to avoid the possibility that sleep restriction would impair the ability to understand the task instructions. Thus, in total three sets of Japanese characters were used. Note that the data from the screening were not included in the analysis. The experiment was designed using Inquisit 4 (www.millisecond.com).

During the test phase, the symbols were mixed, making up 15 combinations that were presented four times, rendering a total of 60 trials. A trial was the same as in the learning phase but without feedback. This phase assesses the ability to generalise. Positive feedback learning is characterised by a higher accuracy for the symbol with highest probability in the learning phase (symbol A) when paired with one of the more neutral stimuli (C, D, E or F) and negative feedback learning by higher accuracy for avoiding the symbol with the lowest probability (symbol B) when paired with the neutral stimuli. Due to the repeated measures design, two different sets of Japanese characters were used and order was counterbalanced between participants.

### 2.4 Statistical analysis

For all analyses, we used Bayesian generalised linear mixed‐effects models (GLMM) fitted in R (R Core Team, 2018). Mixed‐effects models are in general preferable to single‐level analyses, but especially on repeated measures (McElreath, 2016). A Bayesian approach to the GLMM provides rich information in the full posterior distribution, as compared to point estimates, and allows evaluation of the null hypothesis (Kruschke & Liddell, 2018).

#### 2.4.1 Behavioural analyses

For all behavioural analyses we used weakly informative priors with a Student’s *t* distribution (*df* = 3, µ = 0, σ = 2.5) on the intercept and slope. A Cauchy prior (location = 0, scale = 1) was used on the standard deviations (*SDs*). Posterior predictive checks were performed to ensure sufficient model requirements. From the posterior distributions we calculated the highest maximum a posteriori probability estimate (MAP), or the mode of the posterior distribution, and 95% highest density intervals (HDI) for each parameter. However, for best understanding of the data and not merely its summary statistics, readers are encouraged to study the full posterior distribution. A region of practical equivalence (ROPE) was used as proxy for the null hypothesis, with limits set to reflect half of a small effect size (Kruschke, 2018). We also estimated Bayes factors, by taking the likelihood ratios of the posterior distribution falling within or outside the ROPE over the prior distribution (null) falling within or outside the ROPE (Makowski et al., 2019). The Bayes factors denote the evidence of the experimental hypothesis over the null hypothesis (BF_10_) or reversed (BF_01_). A BF_10_ >1 or BF_01_ <1 indicates evidence for the experimental and BF_10_ <1 or BF_01_ >1 indicates evidence for the null hypothesis, with level of evidence considered moderate if above 3 or below 1/3, strong if above 10 or below 1/10, and extreme if above 100 or below 1/100 (Beard et al., 2016).

#### 2.4.2 Computational model

For the computational model we used a Q‐learning algorithm, which estimates the trial‐by‐trial expected value update based on the feedback given (Sutton & Barto, 2018). The analytic procedure were based on that of McCoy et al., (2019), and we adapted the scripts to model sleep as a within subject parameter (scripts available on https://osf.io/mtszr/). The outcome parameters consisted of two learning rates (α), for positive and negative feedback respectively, and inverse temperature (β) reflecting the consistency in the choices. Weakly informative normally distributed priors (µ = 0, σ = 1) were set on the group level and individual level means and half‐Cauchy priors (location = 0, scale = 5) were set on the group level *SD*s. Q‐values were initialised at 0.5. Parameters were transformed to an inverse probit distribution and centred on zero with a *SD* of 1 and with restrictive boundaries of ± 5 (see Ahn et al., 2017). We fitted one model with data from all learning blocks and one with data from the first learning block only. For evaluative purposes, we also fitted models with a single learning rate parameter (positive and negative combined), although they did not provide a better fit than the models with two learning rate parameters evaluated by leave‐one‐out cross validation (LOO; Vehtari et al., 2017) (see Table S9).

For further details on the statistical analysis we refer to the Appendix S1.

## 3 RESULTS

### 3.1 Sleepiness increased

**FIGURE 2:**
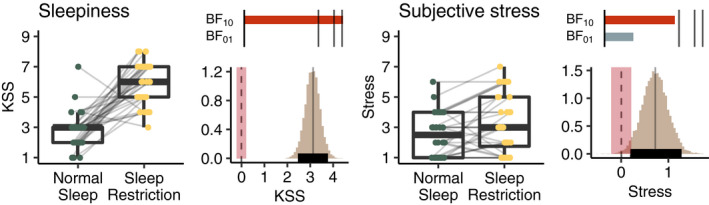
Boxplots show observed sleepiness ratings according to the Karolinska Sleepiness Scale (KSS; Åkerstedt &amp; Gillberg, 1990), and subjective stress ratings (Schwarz et al., 2018) for the normal and restricted sleep conditions. Histograms to the right of each boxplot show the posterior distributions of the difference between sleep conditions with highest density intervals (HDI; thick black horizontal line), highest maximum a posteriori probability estimates (MAP; grey solid vertical line), and the regions of practical equivalence (ROPE; red shading) around zero (dotted line). Sleepiness increased strongly but an increase in stress after sleep restriction was not large enough to be conclusively separated from the ROPE. Bars above the histograms show Bayes factors with level of support for either hypothesis (BF_10_, red; BF_01_, grey) indicated by length of the bar; black lines indicate thresholds for moderate (BF >3), strong (BF >10), and extreme evidence (BF >100) (Beard et al., 2016)

Participants were markedly sleepier after 2 nights of sleep restriction as indicated by extreme evidence for an increase on the KSS (Åkerstedt & Gillberg, 1990) (observed mean increase in sleepiness = 3.12, *SD* = 2.14, MAP = 3.11, 95% HDI = 2.47–3.78, BF_10_ = 1e + 10, BF_01_ = 8e − 11). A small increase in subjective stress (Schwarz et al., 2018) (mean increase in stress = 0.72, *SD* = 1.51) was, however, not distinct enough to reach the criterion of moderate evidence (MAP = 0.72, 95% HDI = 0.19–1.28, BF_10_ = 2.45, BF_01_ = 0.41). For full posterior distributions and observed data see Figure 2.

### 3.2 Sleep restriction did not affect win–stay/lose–shift tendencies

**FIGURE 3:**
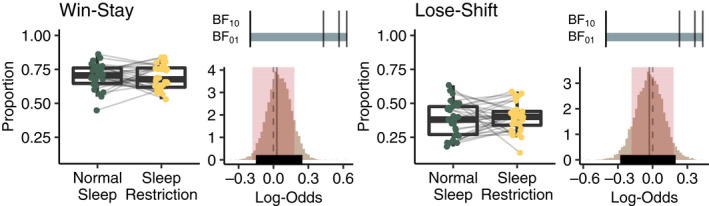
Boxplots show observed data for win–stay and lose–shift tendencies during the first block of the learning phase. Histograms to the right of each boxplot show the posterior distributions of the difference between sleep conditions with highest density intervals (HDI; thick black horizontal line), highest maximum a posteriori probability estimates (MAP; grey solid vertical line) and the regions of practical equivalence (ROPE; red shading) around zero (dotted line), indicating no meaningful difference between sleep conditions. Bars above the histograms show Bayes factors with level of support for either hypothesis (BF_10_, red; BF_01_, grey) indicated by length of the bar and black lines indicate thresholds for moderate (BF >3), strong (BF >10), and extreme evidence (BF >100) (Beard et al., 2016)

To investigate short‐term learning, we calculated the proportion of trials where the choice was to select the same symbol that rendered positive feedback on the previous trial (win–stay) and the proportion of trials where the choice was to switch when given negative feedback (lose–shift). In the first block of the learning phase sleep restriction did not affect the proportion of win–stay (MAP = 0.01, 95% HDI = −0.03 to 0.05, BF_10_ = 0.008, BF_01_ = 127) or lose–shift (MAP = −0.01, 95% HDI = −0.07 to 0.05, BF_10_ = 0.009, BF_01_ = 108) tendencies. In fact, the data suggest extreme evidence for no meaningful difference between the sleep conditions (see also Figure 3). When averaging the data over the two sleep conditions, the proportion of win–stay was higher compared to lose–shift (MAP = 0.30, 95% HDI = 0.26–0.34, BF_10_ = 7e + 12, BF_01_ = 1e − 13).

In addition, we investigated the number of blocks needed to pass the learning criteria. Fewer individuals in the sleep restriction condition passed the criteria before the six‐block limit (26 vs. 30). Here we found that the predicted mean probability of passing the criteria after partial sleep deprivation (0.92) was slightly lower compared to after normal sleep (0.98) with BF_10_ = 7.79, BF_01_ = 0.13 (Figure S1). The average number of blocks needed to pass showed no meaningful difference between the sleep conditions (BF_10_ = 0.23, BF_01_ = 4.26). See Appendix S1 for details. We also performed a supplementary analysis on response times during the learning phase. These showed no difference between sleep conditions (Figure S4).

### 3.3 Sleep restriction did not affect generalized learning incentives

**FIGURE 4:**
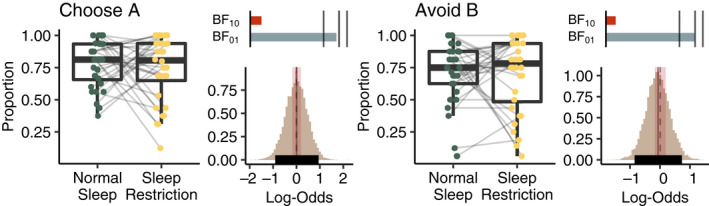
Boxplots of the observed test phase data for Choose A (positive feedback) and Avoid B (negative feedback). Histograms to the right of each boxplot show the posterior distributions of the difference between sleep conditions with highest density intervals (HDI; thick black horizontal line), highest maximum a posteriori probability estimates (MAP; grey solid vertical line) and the regions of practical equivalence (ROPE; red shading) around zero (dotted line). There was no meaningful difference in generalised learning after sleep restriction. Bars above the histograms show Bayes factors with level of support for either hypothesis (BF_10_, red; BF_01_, grey) is indicated by the length of the bar and black lines indicate thresholds for moderate (BF >3), strong (BF >10), and extreme evidence (BF >100) (Beard et al., 2016)

In the test phase, we investigated how the participants had generalised the learning from the learning phase. We were specifically interested in whether learning was prioritised for positive or negative feedback. Positive feedback learning is indicated by the proportion of choices of the symbol with highest probability to win (A) over the more neutral symbols (C, D, E, F) and negative feedback learning is indicated by the proportion where the choice was not to select the symbol with least probability to win (B) when paired with the more neutral symbols. Here we also did not observe any differences between sleep conditions in the accuracy for positive (MAP = 0.0, 95% HDI = −0.10 to 0.11, BF_10_ = 0.13, BF_01_ = 7.54) or negative (MAP = −0.02, 95% HDI = −0.12 to 0.10, BF_10_ = 0.11, BF_01_ = 9.39) feedback learning. Instead, our data are favour the null hypothesis, that learned associations are practically equal for both positive and negative feedback after sleep restriction compared to normal sleep (Figure 4). Collapsing over sleep conditions there was no difference in learning accuracy between positive and negative feedback (MAP = 0.07, 95% HDI = −0.01 to 0.14, BF_10_ = 0.26, BF_01_ = 3.87).

To investigate whether individuals that did not reach the learning criteria before the six‐block limit would bias the results, we also ran the same analysis excluding the individuals that did not pass the learning criteria within the six blocks. This did not change the results (see Appendix S1). Like for the learning phase we analysed the response times during the test phase, including Choose A and Avoid B as a fixed parameter. There was no effect of sleep restriction or symbol pair on response times (Figure S9).

### 3.4 Slower learning rate for negative feedback after sleep restriction

**FIGURE 5:**
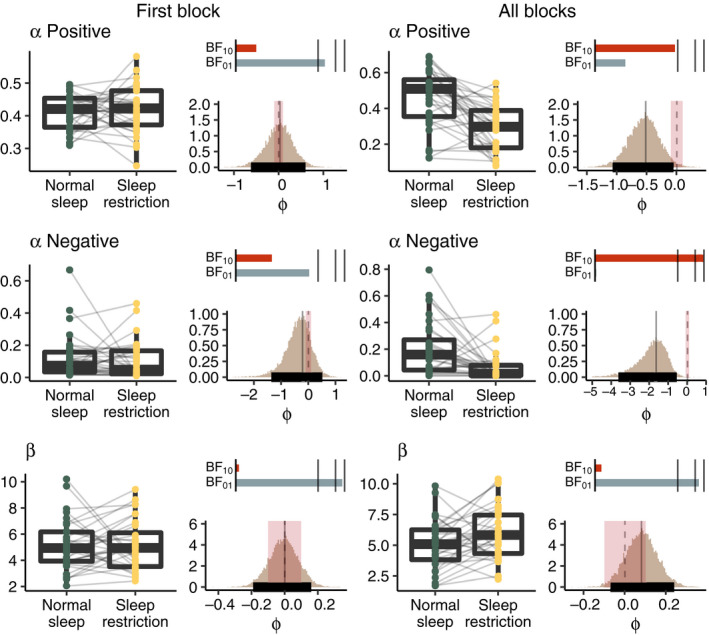
Posterior distributions of the computational model for the first learning block (left panel) and all learning blocks together (right panel). Boxplots show the estimated individual means drawn from the posterior distribution. Histograms to the right of each boxplot show the inverse probit transformed (φ) posterior distributions with highest density intervals (HDI; thick black horizontal line), highest maximum a posteriori probability estimates (MAP; grey solid vertical line) and the regions of practical equivalence (ROPE; red shading) around zero (dotted line). Bars above the histograms show Bayes factors with level of support for either hypothesis (BF_10_, red; BF_01_, grey) indicated by length of the bar and black lines indicate thresholds for moderate (BF >3), strong (BF >10), and extreme (BF >100) evidence (Beard et al., 2016)

To study the trial‐by‐trial learning rate during the learning phase we fitted computational models utilising a Q‐learning algorithm for the first block only, as well as all learning blocks together. See Figure 5 for visualisation of the results. The posterior distribution of the positive learning rate indicated no effect of sleep restriction after the first block (MAP = 0.03, 95% HDI = −0.62 to 0.60, BF_10_ = 0.23, BF_01_ = 4.29), and anecdotal evidence for slower learning after sleep restriction when evaluating all learning blocks (MAP = −0.52, 95% HDI = −1.07 to −0.05, BF_10_ = 2.66, BF_01_ = 0.38). For the negative learning rate, there was anecdotal evidence for no effect of sleep restriction after the first block (MAP = −0.21, 95% HDI = −1.36 to 0.52, BF_10_ = 0.49, BF_01_ = 2.02). However, when considering all learning blocks, there was strong evidence for slower learning after sleep restriction (MAP = −1.65, 95% HDI = −3.63 to −0.56, BF_10_ = 82.44, BF_01_ = 0.01). To further assess the influence of sleep restriction on positive and negative learning rate, we first calculated the difference in posterior distributions between the positive and the negative learning rate (Positive–Negative) stratified by sleep condition. Then we calculated the differences of these differences to estimate whether sleep restriction affects positive and negative learning rate differentially. Results showed inconclusive evidence both for the data from the first block (MAP = 0.95, 95% HDI = −0.49 to 2.38, BF_10_ = 1.88, BF_01_ = 0.53) and for the data from all blocks (MAP = 0.15, 95% HDI = −2.27 to 1.61, BF_10_ = 0.88, BF_01_ = 1.14). For the choice consistency (β), indicating to what extent the individual explored or exploited the options based on the feedback given, there was no difference neither after the first block (MAP = −0.002, 95% HDI = −0.19 to 0.16, BF_10_ = 0.03, BF_01_ = 34.40) nor after all blocks (MAP = 0.09, 95% HDI = −0.07 to 0.24, BF_10_ = 0.06, BF_01_ = 17.17).

## 4 DISCUSSION

In the present study, 2 nights of sleep restriction increased sleepiness, but did not affect generalised learning in the test phase of the reinforcement learning task. Utilising Bayesian statistics with Bayes factors showing the probability of one hypothesis over the other, we found that the data favoured the null hypothesis, indicating that participants in both sleep conditions learned equally well from positive and negative feedback. There was no support for sleep restriction having an effect on the neither the win–stay nor lose–shift learning strategies over the first 60 trials of the learning phase. Complementary computational modelling using a Q‐learning algorithm further suggested that learning rate, estimated from the trial‐by‐trial behaviour in the first block of the learning phase did not differ between sleep conditions. However, when evaluating all learning phase blocks, there was strong evidence of slowed learning rate for negative feedback after sleep restriction.

We did not observe alterations in generalised learning or win–stay/lose–shift tendencies after sleep restriction, as indicated by the null hypothesis being between seven and >100 times more likely than the experimental hypothesis. Most of the previous research is based on total sleep deprivation (Liu & Zhou, 2016; Whitney et al., 2015) and it is possible that 2 nights of sleep restriction did not have the same effect on reward incentives. It has been hypothesised that adenosine accumulation is involved in the dopamine alterations (Krause et al., 2017), and this accumulation could possibly have been restored enough from the, although short, sleep period (Elmenhorst et al., 2017). Another reason for the lack of effect of sleep restriction on reinforcement learning could be that the probabilistic selection task was not sensitive enough to capture such changes after sleep restriction. On the other hand, other studies using a similar assumption regarding dopamine availability, such as Parkinson’s disease (Frank, 2004; Frank et al., 2007; McCoy et al., 2019) and stress (Lighthall et al., 2013; Petzold et al., 2010), and the same reinforcement learning task, have found an effect on generalised learning. Although the probabilistic selection task is a well‐established paradigm, the feedback may not have triggered a hedonic reward signal comparable to those of economic, food or pleasurable images used in previous sleep deprivation studies (Greer et al., 2013; Gujar et al., 2011; Mullin et al., 2013; Venkatraman et al., 2007). Thus, the reward in our paradigm was potentially too weak or not valued relevant enough to cause detectable behavioural changes after sleep restriction. An avenue for further studies is to investigate the effect of total sleep deprivation on reinforcement learning, possibly combined with more ecologically valid or stronger reinforcers in relation to sleep loss.

The results from the computational model suggest that the trial‐by‐trial negative learning rate is affected by sleep restriction when evaluating the full learning phase, but not when only evaluating data from the first block. The win–stay and lose–shift reflected the total proportions of respective behaviour for the first learning phase block, and only considered the response of the previous trial. The Q‐learning estimates, on the other hand, was based on the trial‐by‐trial value update that considers the continuous learning from all previous feedback. For the first block, the results were somewhat in agreement with the win–stay and lose–shift results, although there is uncertainty in the effect of sleep restriction for learning rate from negative feedback. For the data from the full learning phase, we observed a slowing of learning rate after sleep restriction for negative feedback, and a slight slowing for positive feedback that was not confidently supported by the Bayes factor (BF_10_ = 2.66). Thus, rather than the hypothesised increase in reward seeking, we observed a slowing of learning speed from negative feedback after sleep restriction that could be related to increased risk‐seeking behaviour found after sleep restriction (Killgore, 2015; Maric et al., 2017). Reinforcement learning algorithms, such as Q‐learning, have been directly linked to dopamine activity (Garrison et al., 2013). Total sleep deprivation has been associated with downregulation of dopamine D_2_ receptor availability (Volkow et al., 2012), and lower D_2_ receptor availability has been linked to slower learning rate specifically for negative feedback (Frank et al., 2007). Slowing in learning rate could indicate problems in maintaining feedback information in working memory and slower integration of information over time (Frank et al., 2007). Sleep restriction caused a slower learning rate for negative feedback, but to some degree also affected the learning rate for positive feedback. Moreover, high uncertainty in the difference estimates between positive and negative learning rate between the two sleep conditions did not confidently favour one hypothesis over the other. This could be an indication of a general working memory decline or attention deficits commonly found after sleep restriction (Lowe et al., 2017) rather than being associated to specific feedback valence. Lastly, sleep restriction did not affect the inverse temperature (β), indicating that the level of consistency in choice behaviour was similar across conditions.

To limit differences between individuals regarding time of task, we used a limit of six blocks for completing the learning phase, regardless of passing the learning criteria or not. After sleep restriction six out of the 32 participants did not reach the criteria within this limit compared to two out of the 32 after normal sleep, a difference with moderate support from the Bayes factor. This could be related to general attentional or working memory impairments after sleep restriction (van Dongen et al., 2003; Lowe et al., 2017), but as this was not a main question of interest further studies are needed to explore the underlying mechanisms for why sleep restriction may reduce the probability of reaching a learning criterion.

There are some limitations in the present study worth mentioning. The sleep manipulation was done in the participants’ homes restricting the possibilities of controlling adherence to the protocol. For the 2 manipulation nights, sleep was measured using actiography and sleep diaries. With a few exceptions, these data show satisfactory adherence to the sleep restriction protocol. For the other nights before and in‐between the test sessions we did not measure sleep but relied on the participants keeping to their habitual sleeping pattern and following the instructions not to take naps during the day. However, we have little reason to believe that any divergence from these patterns would be other than random, therefore not changing the conclusions of the study. Finally, these results should be interpreted with the notion that 2 nights of sleep restriction may not be directly transferable to potential effects of total sleep deprivation. Sleep restriction may be a more ecologically valid form of sleep loss, and similar deficits have been found for executive functions and vigilant attention (van Dongen et al., 2003; Lowe et al., 2017), yet less is known about underlying reward incentives after sleep restriction.

To conclude, 2 nights of sleep restriction did not affect the accuracy in generalised learning from positive or negative feedback, the win–stay/lose–shift tendencies, and the modelled learning rate in the initial learning phase. However, considering all blocks from the learning phase using a computational modelling approach revealed that sleep restriction causes a slower learning rate for negative feedback.

## CONFLICT OF INTEREST

The authors declare no competing interests.

## AUTHOR CONTRIBUTIONS

Conceptualisation: JS, AG and JA; Methodology: JS, AG and JA; Investigation: DKP and JNL; Analysis: AG, JS and JA; Interpretation: AG, JS and JA; Writing – original draft: AG; Writing – Review and Editing: AG, JS, JA, JNL and DKP; Supervision: JS, JA and JNL.

## Supporting information

Appendix S1

## Data Availability

Data and analysis scripts are available via Open Science Framework (https://osf.io/mtszr/).
