# Supplementary material for: Does insufficient sleep affect how you learn from reward or punishment? Reinforcement learning after 2 nights of sleep restriction
Source: J Sleep Res. 2020 Nov 20;30(4):e13236. doi: 10.1111/jsr.13236 (PMC8365707; doi:10.1111/jsr.13236)
Supplement: Supplementary file 1 — Appendix S1 [file JSR-30-e13236-s001.pdf]

Supplementary material

Title: Does insufficient sleep affect how you learn from reward or punishment? – Reinforcement learning after two nights of sleep restriction

*Andreas Gerhardsson[1,2], Danja K. Porada[3], Johan N. Lundström[3,4,5,6], John Axelsson[1,2,3] & Johanna Schwarz[2,3]

[1] Department of Psychology, Stockholm University, Stockholm, Sweden
[2] Department of Psychology, Stress Research Institute, Stockholm University, Stockholm, Sweden
[3] Departement of Clinical Neuroscience, Karolinska Institute, Stockholm, Sweden
[4] Monell Chemical Senses Center, Philadelphia, Pennsylvania
[5] Department of Psychology, University of Pennsylvania, Philadelphia
[6] Stockholm University Brain Imaging Centre, Stockholm University, Stockholm, Sweden

* Corresponding author:
Andreas Gerhardsson
Department of Psychology, Stockholm University
andreas.gerhardsson@su.se

1 Methods

1.1 Participants

To measure visual and auditory acuity we used Snellen's visual acuity evaluation (Snellen, 1862) and a computerized whispered voice test (Pirozzo, 2003). Wash-out period between the tests was 7-8 days for all participants except two for whom the period was 12 and 17 days. Two participants stand-out with wake-up times on the day of the normal sleep test session at around 09:30. To evaluate the influence of delayed wake-up times and the fact that test times differed between individuals we ran supplementary analyses including hours awake before test session (test time - sleep-end) as population level and group varying effect (see sections 3.2 and 4). Although hours awake had some influence on the results, keeping the variable constant did not change the conclusions of no meaningful difference between the sleep conditions.

1.2 Statistical analyses details

For all statistical analyses we used Stan (Stan Development Team, 2018) via R (R Core Team, 2018) to fit Bayesian generalized linear mixed-effects models (GLMM) using Markov Chain Monte Carlo (MCMC) sampling. For all behavioral analyses we used the brms-package (Bürkner, 2017).

Weakly informative priors, intended to aid the model fitting but to have little weight on the posterior, were set on the intercept and fixed coefficients to a student t-distribution with 3 degrees of freedom, a mean of 0 and standard deviation of 2.5 (Ghosh et al., 2017), and a Cauchy prior on the sigma with a scale of 1. All model predictors were dummy coded and centered around zero. Posterior distributions, the highest maximum a posteriori probability estimate (MAP) and 95% highest density intervals (HDI) were calculated for each parameter, together with a Bayes Factors. The MAP and the HDI summarizes the peak and the uncertainty in the measure by indicating the most probable values given the data (Kruschke, 2015). To directly test the hypotheses, we calculated Bayes Factors (Makowski et al., 2019) – likelihood ratios of the experimental over the null hypothesis BF_{10} and reversed ($BF_{01} = 1/ BF_{10}$). A region of practical equivalence (ROPE), was used as proxy for the null hypothesis with limits set to reflect half of a small effect size (Kruschke, 2018). A $BF_{10} > 1$ or $BF_{01} < 1$ indicates evidence for the experimental and $BF_{10} < 1$ or $BF_{01} > 1$ indicates evidence for the null hypothesis, with level of evidence considered moderate if above 3 or below 1/3, strong if above 10 or below 1/10, and extreme if above 100 or below 1/100 (Beard et al., 2016).

2 Sleepiness and stress

To analyze sleepiness according to the Karolinska Sleepiness Scale (KSS: Åkerstedt and Gillberg, 1990) and self-rated stress (see Table S1 for observed data), we fitted a Bayesian GLMM with a gaussian link function. Priors were set to a student-*t* distribution with df = 3, mu = 0 and sigma = 2.5 on the intercept and slope. A Cauchy prior with location = 0 and scale 1 was used on the SD. We used 4 chains with 4000 iterations (including 1000 for warm-up). Posterior predictive checks indicated no divergence and no parameters exceeding Rhat limit (< 1.1). The model was fitted with KSS and stress as dependent variables respectively, sleep (centered) as within participant predictor and a varying intercept for each participant. Model estimations was drawn from the posterior distributions (Table S2).

Table S1. Observed data of KSS and stress measured before the probabilistic selection task.

		KSS	Stress
Sleep condition	*n*	*Mean (SD)*	*Mean (SD)*
Normal Sleep	32	2.84 (1.19)	2.53 (1.41)
Sleep Restriction	32	5.97 (1.40)	3.25 (1.90)
Δ	32	3.12 (2.14)	0.72 (1.51)

Table S2. Model estimates of KSS and stress from the posterior distributions with standard error,

	Mean estimate	*Error estimate*	*Q 2.5*	*Q 97.5*	*MAP estimate*	*HDI 2.5*	*HDI 97.5*	BF_{10}	BF_{01}
KSS									
Normal Sleep	2.84	0.24	2.38	3.30	2.84	2.38	3.31		
Sleep Restriction	5.95	0.24	5.46	6.43	5.94	5.44	6.40		
Δ	3.11	0.33	2.45	3.76	3.13	2.47	3.78	1e+10	8e-11
Stress									
Normal Sleep	2.50	0.31	1.88	3.11	2.51	1.87	3.10		
Sleep Restriction	3.21	0.31	2.59	3.82	3.23	2.62	3.84		
Δ	0.72	0.28	0.17	1.26	0.72	0.19	1.28	2.45	0.41

3 Learning phase

3.1 Number of blocks

To leave the learning phase and proceed to the test phase, the participants had to reach the learning criteria for all the symbol pairs after a block (≥ 65% A choices for A/B trials, ≥ 60% C choices for C/D trials and ≥ 40% E choices for E/F trials). However, to reduce the time on task we restricted the maximum number of learning blocks to 6. After normal sleep, 2 out of 32 individuals did not reach the criteria and after sleep restriction 6 out of 32 individuals did not reach the criteria. The mean number of blocks needed to reach the learning criteria (excluding the individuals that failed to reach the criteria) was 1.97 ± 1.07 after normal sleep and 1.85 ± 1.41 after sleep restriction.

To analyze the number of blocks completed in the learning phase (see Table S3 for observed data) we fitted a Bayesian generalized linear censored (Hilbe, 2011) model with a *Poisson* family log-link function. The censoring was applied due to the maximum possible number of blocks were limited to 6, thus the *true* number of blocks needed to reach learning criteria was unknown. Priors were set to a student-*t* distribution with df = 3, mu = 0 and sigma = 2.5 on the intercept and slope. A Cauchy prior with location = 0 and scale 1 was used on the SD. We used 4 chains with 4000 iterations (including 1000 for warm-up). Posterior predictive checks indicated no divergence and no parameters exceeding Rhat limit (< 1.1). After comparing different models, the best fitted model included number of blocks as dependent variable and passing the criteria as censored variable, sleep (centered) as within participant predictor order as between participant covariate and a varying intercept and slope for each participant by sleep condition. Model estimations was drawn from the posterior distributions (Table S4 and Figure S1). In addition, we analyzed if the number of individuals passing the criteria differed, using a Bayesian GLMM with a Bernoulli family log-link function with the same priors and MCMC settings as in the number of blocks model. The posterior distributions predicted a decrease in the probability of passing the criteria after sleep restriction (Table S4 and Figure S1).

Table S3. Observed number of learning blocks and number of participants passing the learning criteria.

	Median	*Mean (SD)*	*N Pass criteria (%)*
All participants			
Normal Sleep	2	2.22 (1.43)	
Sleep Restriction	1.5	2.62 (2.08)	
Δ	-0.5	0.41 (1.72)	
Passing criteria			
Normal Sleep	2	1.97 (1.07)	30 (93.75)
Sleep Restriction	1	1.85 (1.41)	26 (81.25)
Δ	-1	-0.12 (1.72)	-4 (-12.50)

Table S4. Model estimates of number of blocks and probability of passing criteria.

	Mean estimate	*Error estimate*	*Q 2.5*	*Q 97.5*	*MAP estimate*	*HDI 2.5*	*HDI 97.5*	*BF_{10}*	*BF_{01}*
Number of blocks									
Normal Sleep	2.02	0.34	1.40	2.71	1.96	1.38	2.69		
Sleep Restriction	2.47	0.51	1.58	3.57	2.29	1.51	3.47		
Δ	-0.44	0.48	-1.42	0.46	-0.37	-1.40	0.47	0.23	4.26
Passing criteria									
Normal Sleep	0.98	0.03	0.90	1.00	1.00	0.92	1.00		
Sleep Restriction	0.92	0.08	0.73	1.00	1.00	0.76	1.00		
Δ	0.06	0.07	0.01	0.23	0.01	-0.0§	0.22	7.79	0.13

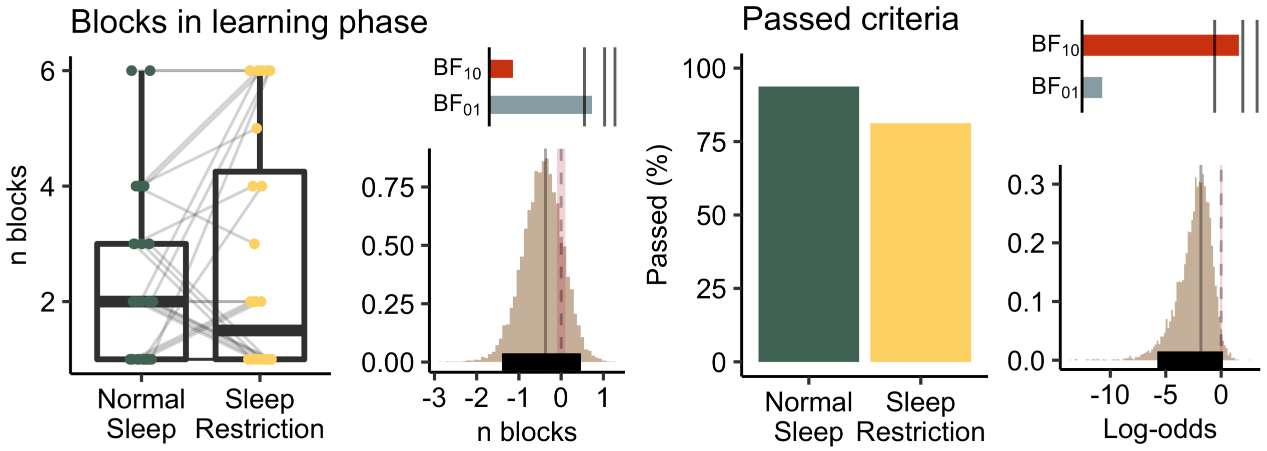

Figure S1. Boxplot with observed number of blocks until reaching learning criteria and bar plot of percent passing criteria per sleep condition. Histogram show posterior distributions of the difference between the sleep conditions, with highest density intervals (HDI; thick black horizontal line), highest maximum a posteriori probability estimates (MAP; grey solid vertical line) and the regions of practical equivalence (ROPE; red shading) including zero (dotted line), supporting no meaningful difference between the sleep conditions. Bars above histogram show Bayes factors with level of support for either hypothesis (BF_{10}: red; BF_{01}: grey) indicated by length of the bar, black vertical lines mark the level of evidence from moderate (BF > 3), strong (BF > 10) to very strong (BF > 100).

3.2 Win-stay/Lose-shift

Win-stay indicates the tendency to select the same symbol that rendered positive feedback from the previously presented (one trial back in the sequence) symbol pair and lose-shift the to select the

opposite symbol as the one leading to negative feedback in the previous symbol pair. Win-stay and lose-shift was coded as binomial (1, 0) outcome variables and a generalized linear model with a *Bernoulli* logit link function was fitted for each outcome. Observed data is presented in Table S5. Priors were set to a student-*t* distribution with df = 3, mu = 0 and sigma = 2.5 on the intercept and slope. A Cauchy prior with location = 0 and scale 1 was used on the SD. We used 4 chains with 4000 iterations (including 1000 for warm-up) for the MCMC sampling. Posterior predictive checks indicated no divergence, no parameters exceeding Rhat limit (< 1.1) and good fit to the data (Figure S2). The models included win-stay or lose-shift as dependent variables, sleep (centered) as within participant predictor, order as between participant covariate and a varying intercept and slope for each participant by sleep condition. Model estimations was drawn from the posterior distributions (Tables S6 and Figure S3). Bayes Factors was estimated from the Log-Odds distributions.

Table S5. Observed means and standard deviations (SD) of win-stay and lose-shift proportions for each sleep condition and the difference between sleep conditions.

	Win-stay		Lose-shift	
	Mean	*SD*	*Mean*	*SD*
Normal Sleep	0.70	0.09	0.39	0.12
Sleep Restriction	0.69	0.09	0.40	0.10
Δ	0.01	0.12	-0.01	0.15

Table S6. Table of proportions estimates, difference between sleep conditions and difference between win-stay and lose shift, collapsed over sleep conditions.

	Mean estimate	*Error estimate*	*Q 2.5*	*Q 97.5*	*MAP estimate*	*HDI 2.5*	*HDI 97.5*	BF_{10}	BF_{01}
Win-stay	0.01	0.02	-0.03	0.05	0.01	-0.03	0.05	0.008	127
Lose-shift	-0.01	0.03	-0.06	0.05	-0.01	-0.07	0.05	0.009	108
Δ	0.30	0.02	0.26	0.34	0.30	0.26	0.34	7e+12	1e-13

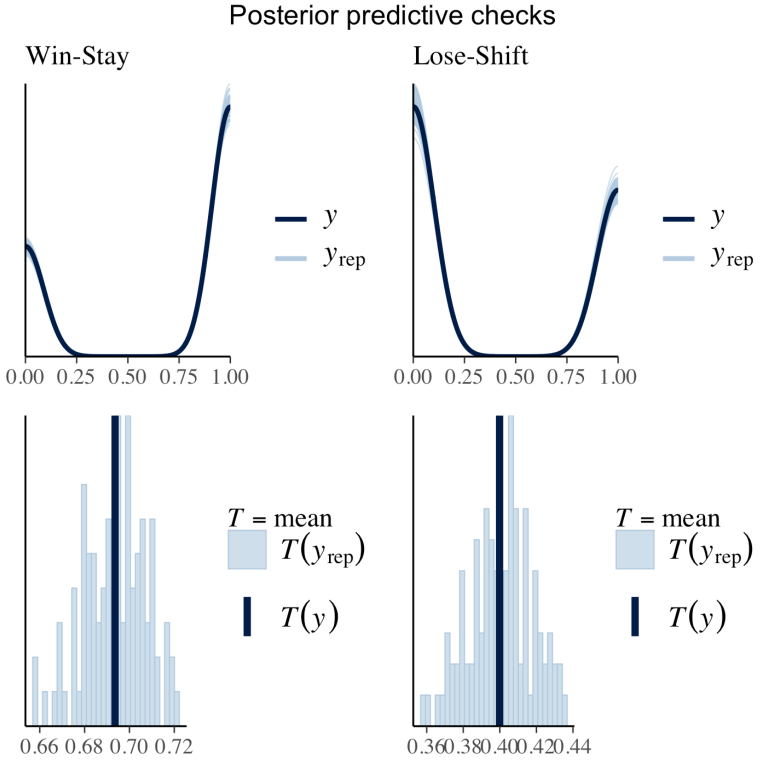

Figure S2. Posterior predictive checks of Win-stay and Lose-shift models, both indicating good fit to the data (y).

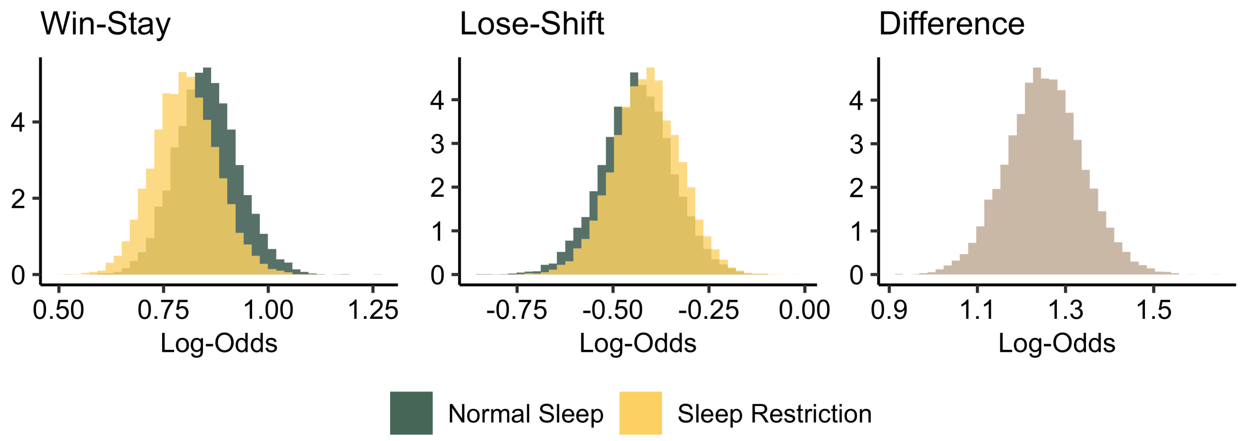

Figure S3. Posterior distributions of Win-stay and Lose-shift tendencies separated by Normal sleep (green) and Sleep restriction (yellow). The right most panel shows the distribution of the difference between Win-stay and Lose-Shift collapsed over sleep condition.

To evaluate the influence of wake-up time, test time and time awake before the test, we fitted a model accounting for time awake before the test (test time – wake-up time) by adding it as a population level effect and a group varying slopes for each participant for win-stay and lose shift. Keeping the time awake constant had no meaningful impact on the results (see Table S7).

Table S7. Table of proportions win-stay and lose-shift estimates from the model adjusted for time before the test.

	Mean estimate	*Error estimate*	*Q 2.5*	*Q 97.5*	*MAP estimate*	*HDI 2.5*	*HDI 97.5*	*BF_{10}*	*BF_{01}*
Win-stay	-0.01	0.03	-0.06	0.05	-0.01	-0.06	0.05	0.012	85
Lose-shift	0.02	0.04	-0.05	0.09	0.02	-0.05	0.09	0.025	40
Δ	0.31	0.02	0.27	0.36	0.31	0.27	0.36	4.e+10	2e-11

3.3 Response times

We also ran an exploratory analysis on response times for the learning phase. First, response times were normalized and centered around zero. Then we fitted a generalized linear model with an ex-gaussian link function to adjust for the expected positive skew. Otherwise the procedure was similar to the Win-stay/Lose-shift modelling. Figure S4 shows the results, indicating no effect of sleep restriction on response times ($BF_{10} = 0.017$, $BF_{01} = 58.59$) in the learning phase.

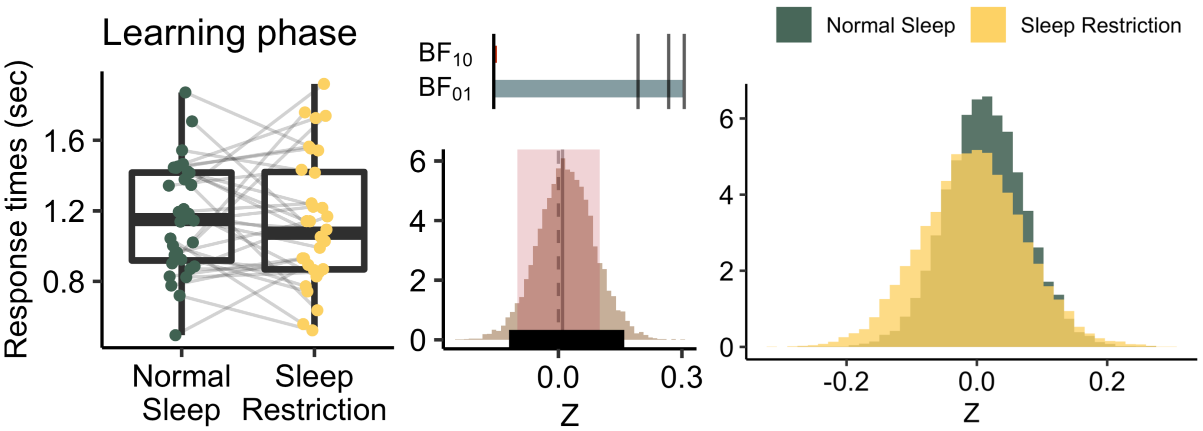

Figure S4. Response times during learning phase. The histogram directly next to the boxplot shows posterior distributions of the difference in response time between the sleep conditions, with highest density intervals (HDI; thick black horizontal line), highest maximum a posteriori probability estimates (MAP; grey solid vertical line) and the regions of practical equivalence (ROPE; red shading) including zero (dotted line), supporting no meaningful difference between the sleep conditions. Bars above histogram show Bayes factors with level of support for either hypothesis (BF_{10}: red; BF_{01}: grey) indicated by length of the bar, black vertical lines mark the level of evidence from moderate (BF > 3), strong (BF > 10) to very strong (BF > 100). Histograms to the right show posterior distribution separated by sleep condition.

3.4 Computational analyses

To estimate trial-by-trial learning behavior we modelled the data from the learning phase using a Q-learning algorithm via rstan (Stan Development Team, 2018). Overall we followed the analytic procedure described in McCoy et al., (2019). Group and individual means were fitted using a weakly informative prior of a normal distribution with a mean of 0 and a standard deviation of 1 and for standard deviations we used half-Cauchy priors (location = 0, scale = 5). Q-values were initialized at 0.5, assuming equal expected value of the symbols at first trial for each pair. Inverse probit transformed parameter estimates are presented in Table S8 and posterior distributions of the sleep conditions for non-transformed data are visualized in Figure S5. We also compared the two learning rates model with a single learning rate model and for both the first block data and all block data the two learning rates model was a better fit than the single learning rate model (Table S9).

Table S8. Parameter, learning rate (α) and inverse temperature (β), estimates of differences between sleep conditions from posterior distributions.

	Mean estimate	*Error estimate*	*Q 2.5*	*Q 97.5*	*MAP estimate*	*HDI 2.5*	*HDI 97.5*	BF_{10}	BF_{01}
First block									
α Positive	-0.017	0.0031	-0.47	0.43	-0.004	-0.45	0.44	0.16	6.20
α Negative	-0.638	0.0097	-1.86	0.27	-0.502	-1.74	0.35	0.93	1.07
β	0.005	0.0013	-0.17	0.18	-0.001	-0.17	0.18	0.03	33.73
All blocks									
α Positive	-0.51	0.0028	-0.943	-0.11	-0.505	-0.92	-0.09	3.94	0.25
α Negative	-1.29	0.0119	-2.875	-0.29	-1.047	-2.76	-0.23	14.51	0.07
β	0.06	0.001	-0.082	0.20	0.061	-0.08	0.2	0.04	26.50

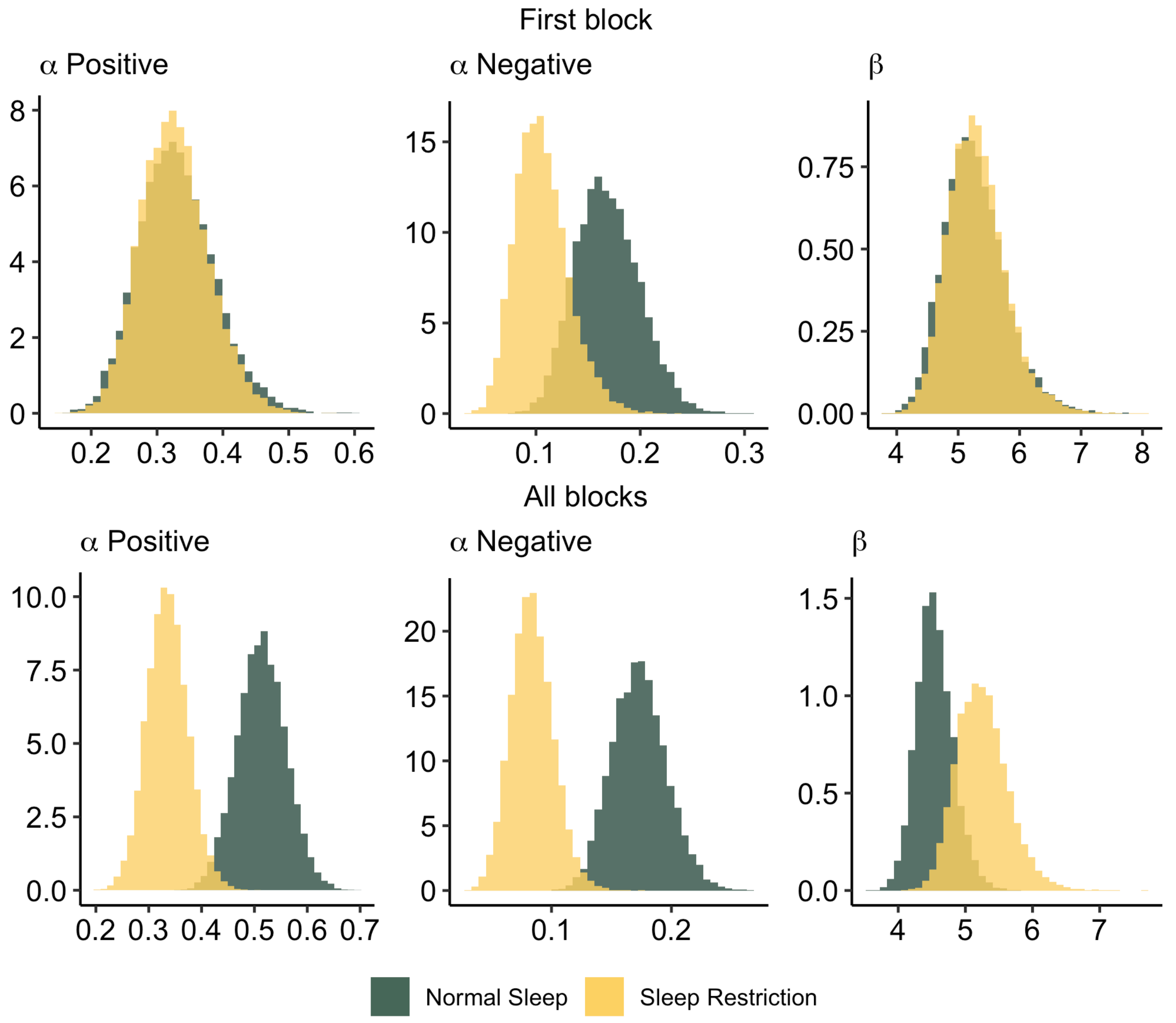

Figure S5. Posterior distribution of computational mode data with Normal sleep (green) and Sleep restriction (yellow). Top panels show data estimated from first learning block and bottom panels show data estimated from all learning blocks.

Table S9. Model comparison of models with single learning rate and two learning rates for data derived from the first learning block or all learning blocks using leave-one-out cross-validation (LOO). Both for first block and all blocks the two-alpha model indicates a better fit as indicated by the difference in ELPD (expected log pointwise estimate). (Vehtari et al., 2020; LOO: Vehtari et al., 2017).

Model	$ELPD_{DIFF}$ (SE)	$ELPD_{LOO}$ (SE)	P_{LOO} (SE)	LOOIC (SE)
Two alpha (First block)	0	-936.54 (51.41)	46.11 (3.21)	1873.08 (102.82)
Single alpha (First block)	-47.38 (15.74)	-983.92 (45.12)	37.7 (3.38)	1967.84 (90.23)
Two alpha (All blocks)	0	-2428.19 (391.39)	60.61 (4.27)	4856.37 (782.78)
Single alpha (All blocks)	-112.27 (39.29)	-2540.45 (402.57)	53.16 (4.61)	5080.9 (805.14)

Note: P_{LOO} = effective number of parameters, LOOIC = leave-one-out information criterion

To further evaluate the predictive quality of the two selected models (with two learning rates) we used the parameters (α Positive, α Negative and β) maximum a posteriori probability (MAP) estimates from each individual's posterior distribution and fitted each parameter in a new model, with the parameter estimate as a dependent variable and a sleep × subject interaction as a fixed predictor. For the learning rates we used a beta family logit link function and for the beta we used a gaussian family link function. From these posterior distributions we sampled 200 datasets and compared each individual's MAP from those data sets with the original model MAP estimate. All correlations were between 0.70 and 1.00 (Figure S6).

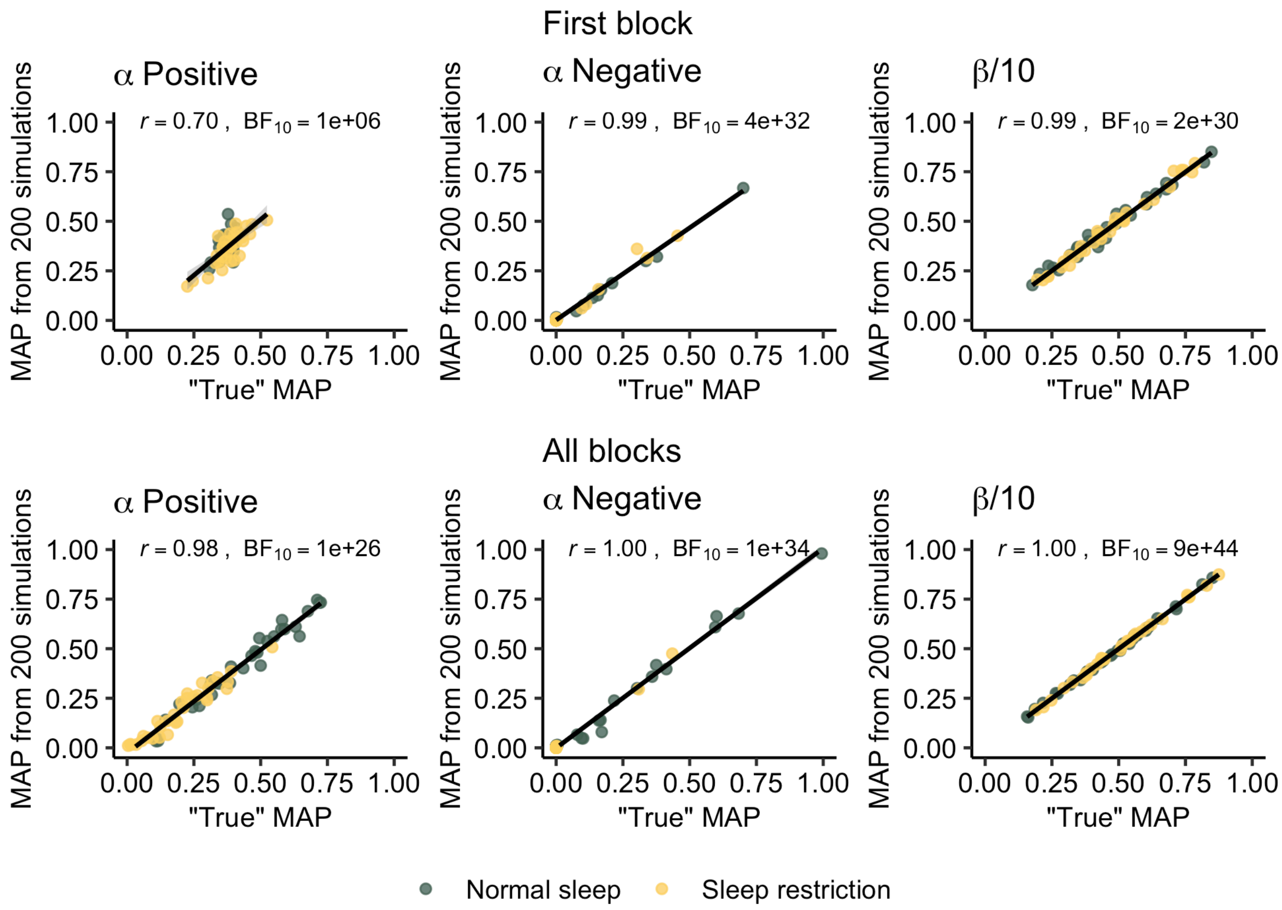

Figure S6. Correlation plots of recovered data for the computational model parameters with maximum a posteriori probability (MAP) estimates from 200 sampled datasets (y-axes) based on the MAP estimate from the original models, the "true" MAP (x-axes). Data points are colored according to sleep condition. The high correlations indicate strong predictability of the data, and Bayes factors (BF_{10}) show extreme evidence in support of the correlation.

To investigated if slower learning rate was associated with the amount of sleep in the two sleep conditions we used Δ assumed sleep (normal sleep – sleep restriction) and model parameters and ran Bayesian correlations analyses using medium-informative priors (r = 0.33). All Bayes factors (BF_{10} [BF_{01}]) where between 0.40 [2.5] and 0.86 [1.16], indicating, if any, anecdotal evidence for the null hypothesis (Figure S7).

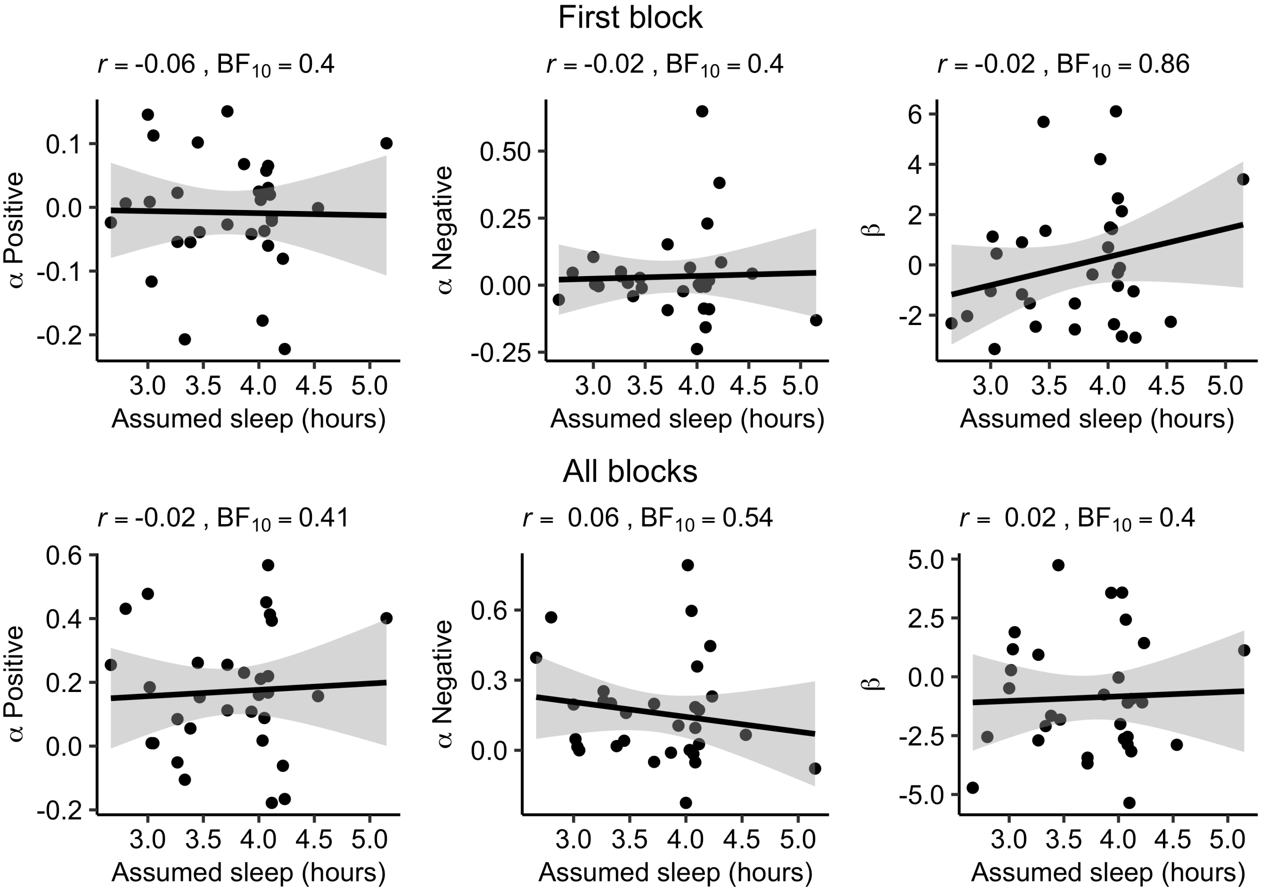

Figure S7. Scatter plots with correlation coefficients and Bayes Factors (BF_{10}) showing the association between Δ assumed sleep (normal sleep – sleep restriction) and computational model parameters for first block (top panel) and all blocks (bottom panel).

4 Test phase

In the test phase a correct response was defined as a response made to the more probable winner symbol in a pair. To measure generalized learning, we used the number of choices made of symbol A, which gave positive feedback 80% of the time when presented paired with more neutral symbols (C, D, E, F) during the learning phase, and the number of choices of the same neutral symbols when paired with the symbol B which gave positive feedback only 20% of the trials during the learning phase. That is, a correct response was to choose symbol A over C, D, E and F and to not choose symbol B over C, D, E and F. Observed data are presented in Table S10. We used a binomial outcome (1, 0) and fitted a Bayesian GLMM with a *Bernoulli* logit link function. Priors were set to a student-*t* distribution with df = 3, mu = 0 and sigma = 2.5 on the intercept and slope. A Cauchy prior with location = 0 and scale 1 was used on the SD. We used 4 chains with 4000 iterations (including 1000 for warm-up) for the MCMC sampling. Posterior predictive checks indicated no divergence, no parameters exceeding Rhat limit (< 1.1) and good fit to data, see Figure S8. The model included correct response as dependent variable, sleep (centered), symbol (centered) and sleep-symbol interaction as within participant predictor, order as between participant covariate and a varying intercept and slope for each participant by sleep, symbol and the interaction. Model estimations was drawn from the posterior distributions (Tables S11). Bayes Factors was estimated from the Log-Odds distributions (Figure S9). Supplementary analysis excluding the individuals that did not reach the criteria after six blocks did not change the results (Tables S12).

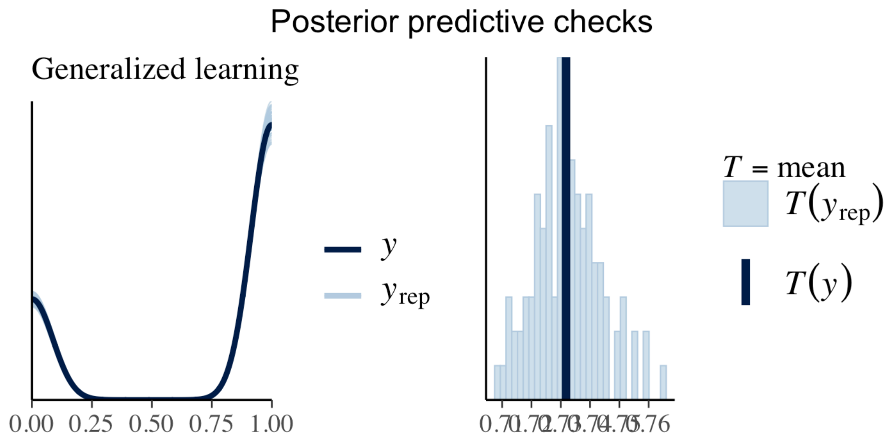

Figure S7. Posterior predictive checks of test phase model and observed data.

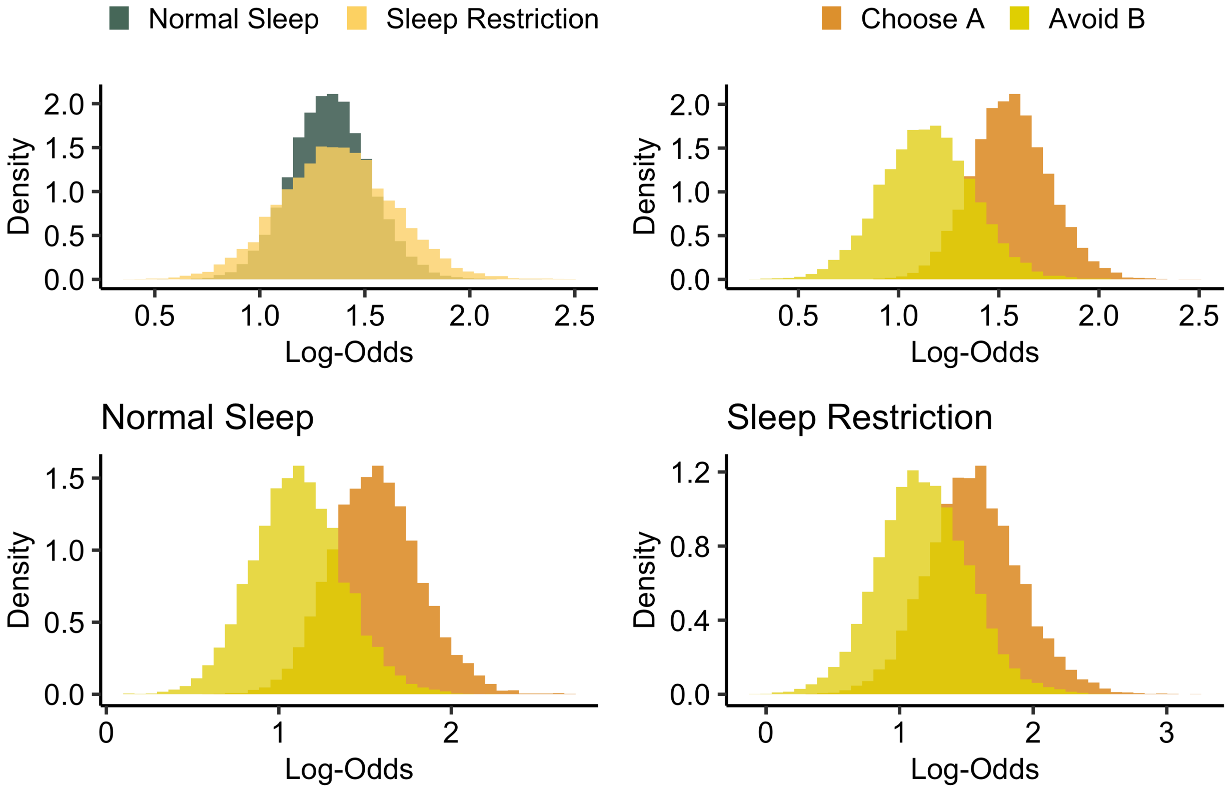

Figure S8. Posterior distributions of test phase data. Top left panel shows distributions separated by sleep condition collapsed over symbol. Top right panel shows the distributions separated by symbol (choose A refers to the proportion of trials when symbol A was chosen over more neutral symbols C, D, E and F; Avoid B refers to the proportion of trials when the more neutral symbols C, D, E, and F were chosen over B) but collapsed over sleep condition. The bottom panels show the distributions separated by symbol and separated by sleep condition.

Table S10. Observed results for generalized learning, where Choose A is the proportion of trials when choosing symbol A over more neutral C, D, E, F, and Avoid B is the proportion of trials choosing symbols C, D, E, and F over B.

		Normal sleep		Sleep restriction	
	n	*Mean (SD)*	*Median*	*Mean (SD)*	*Median*
Choose A	32	0.78 (0.18)	0.81	0.74 (0.25)	0.81
Avoid B	32	0.71 (0.23)	0.75	0.69 (0.28)	0.78

Table S11. Test phase proportion estimates from the posterior distributions.

Estimate	Mean estimate	Error estimate	Q 2.5	Q 97.5	MAP estimate	HDI 2.5	HDI 97.5	BF_{10}	BF_{01}
Normal sleep									
Choose A	0.82	0.04	0.75	0.89	0.83	0.77	0.88		
Avoid B	0.75	0.05	0.65	0.84	0.76	0.67	0.83		
Sleep restriction									
Choose A	0.82	0.05	0.71	0.91	0.83	0.74	0.90		
Avoid B	0.76	0.06	0.63	0.87	0.76	0.67	0.85		
Contrasts									
Δ Sleep	0.00	0.05	-0.10	0.10	-0.01	-0.09	0.08	0.08	12.17
Δ Symbol	0.07	0.05	-0.02	0.16	0.07	-0.01	0.14	0.26	3.87
Δ Sleep | Choose A	0.00	0.07	-0.12	0.14	0.00	-0.10	0.11	0.13	7.54
Δ Sleep | Avoid B	-0.01	0.07	-0.15	0.14	-0.02	-0.12	0.10	0.11	9.39
Δ (Full interaction)	0.01	0.09	-0.17	0.19	0.03	-0.12	0.17	0.17	5.82

Table S12. Test phase proportion estimates from the posterior distributions with individuals not passing criteria excluded.

Estimate	Mean estimate	Error estimate	Q 2.5	Q 97.5	MAP estimate	HDI 2.5	HDI 97.5	BF_{10}	BF_{01}
Normal sleep									
Choose A	0.82	0.04	0.74	0.89	0.83	0.76	0.88		
Avoid B	0.75	0.05	0.65	0.84	0.76	0.68	0.83		
Sleep Restriction									
Choose A	0.83	0.05	0.71	0.92	0.84	0.75	0.91		
Avoid B	0.82	0.05	0.71	0.90	0.82	0.74	0.89		
Contrasts									
Δ Sleep	-0.03	0.06	-0.14	0.08	-0.04	-0.12	0.05	0.12	8.62
Δ Symbol	0.04	0.04	-0.04	0.13	0.03	-0.03	0.11	0.11	9.02
Δ Sleep | Choose A	-0.01	0.07	-0.14	0.14	-0.02	-0.12	0.10	0.14	7.20
Δ Sleep | Avoid B	-0.06	0.07	-0.19	0.07	-0.06	-0.17	0.04	0.18	5.44
Δ (Full interaction)	0.05	0.08	-0.1	0.22	0.05	-0.07	0.19	0.19	5.38

To evaluate the influence of wake-up time, test time and time awake before the test, we fitted a model taking time awake before the test (test time – wake-up time) into account by adding it as a

population level effect and a group varying slopes for each participant. The biggest change can be observed for Avoid B after sleep restriction, although keeping the variable constant had little impact on the overall results. (see Table S13).

Table S13. Proportion of Choose A and Avoid B estimates from the posterior distribution derived from the model controlling for time awake before test.

Estimate	Mean estimate	Error estimate	Q 2.5	Q 97.5	MAP estimate	HDI 2.5	HDI 97.5	BF_{10}	BF_{01}
Normal sleep									
Choose A	0.82	0.05	0.72	0.90	0.84	0.75	0.90		
Avoid B	0.72	0.06	0.58	0.83	0.73	0.62	0.82		
Sleep Restriction									
Choose A	0.81	0.06	0.68	0.9	0.82	0.71	0.89		
Avoid B	0.80	0.05	0.7	0.89	0.82	0.73	0.88		
Contrasts									
Δ Sleep	-0.04	0.06	-0.15	0.09	-0.03	-0.13	0.06	0.11	8.98
Δ Symbol	0.05	0.05	-0.03	0.15	0.06	-0.02	0.13	0.14	6.96
Δ Sleep | Choose A	0.02	0.08	-0.14	0.19	0.01	-0.12	0.15	0.14	7.21
Δ Sleep | Avoid B	-0.09	0.08	-0.26	0.07	-0.07	-0.22	0.04	0.16	6.44
Δ (Full interaction)	0.11	0.11	-0.11	0.33	0.10	-0.07	0.28	0.17	6.03

4.1 Response times

We explored the response times in the test phase using the same setup as for the response times in the learning phase but included *Chose A-Avoid B* as a fixed parameter. Response times were normalized and centered around zero and an exgaussian link function applied to the generalized linear model. Figure S9 show the results and just like the learning phase there was no effect of sleep restriction on the response times. See Table S14 for posterior estimates.

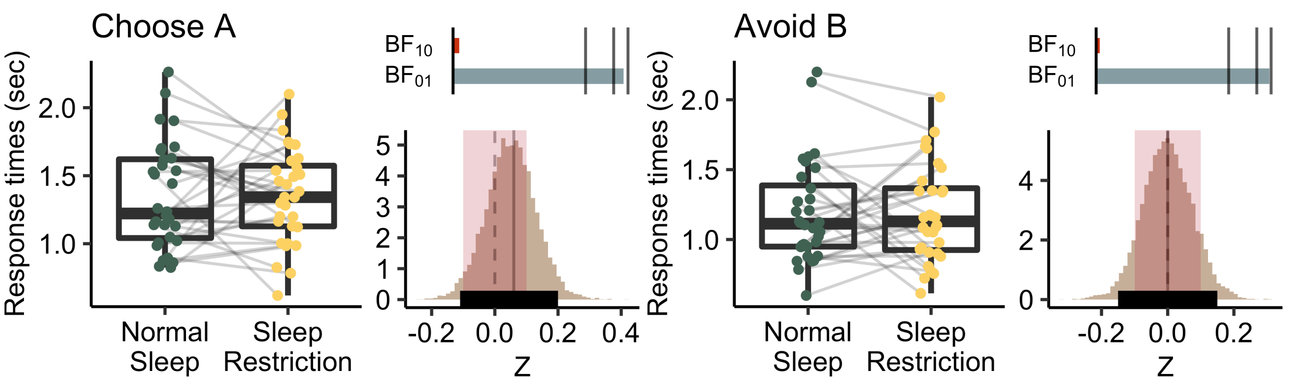

Figure S9. Response times during test phase separate for Choose A and Avoid B. Histogram next to the boxplot show posterior distributions of the response time difference between the sleep conditions, with highest density intervals (HDI; thick black horizontal line), highest maximum a posteriori probability estimates (MAP; grey solid vertical line) and the regions of practical equivalence (ROPE; red shading) including zero (dotted line), supporting no meaningful difference

between the sleep conditions. Bars above histogram show Bayes factors with level of support for either hypothesis (BF_{10}: red; BF_{01}: grey) indicated by length of the bar, black vertical lines mark the level of evidence from moderate (BF > 3), strong (BF > 10) to very strong (BF > 100). Histograms to the right show posterior distribution by sleep condition.

Table S14. Posterior estimates of standardized response times.

Estimate	Mean estimate	Error estimate	Q 2.5	Q 97.5	MAP estimate	HDI 2.5	HDI 97.5	BF_{10}	BF_{01}
Normal sleep									
Choose A	0.06	0.08	-0.10	0.22	0.07	0.07	0.09		
Avoid B	-0.04	0.08	-0.19	0.11	-0.04	-0.05	-0.04		
Sleep Restriction									
Choose A	0.01	0.07	-0.12	0.15	0.02	0.01	0.02		
Avoid B	-0.04	0.07	-0.18	0.10	-0.05	-0.05	-0.03		
Contrasts									
Δ Sleep	0.03	0.07	-0.11	0.17	0.02	0.01	0.03	0.02	50.66
Δ Symbol	0.08	0.03	0.01	0.15	0.08	0.08	0.08	0.029	34.25
Δ Sleep | Choose A	0.05	0.08	-0.10	0.21	0.06	0.06	0.08	0.035	28.40
Δ Sleep | Avoid B	0.00	0.08	-0.15	0.15	0.00	-0.01	0.01	0.019	52.27
Δ (Full interaction)	0.05	0.06	-0.07	0.17	0.05	0.05	0.07	0.023	43.19

5 References

Åkerstedt, T., Gillberg, M. Subjective and objective sleepiness in the active individual. The International journal of neuroscience, 1990, 1: 29–37.

Beard, E., Dienes, Z., Muirhead, C., West, R. Using Bayes factors for testing hypotheses about intervention effectiveness in addictions research: Using Bayes factors for testing hypotheses about intervention effectiveness in addictions research. Addiction, 2016, 111: 2230–2247.

Bürkner, P.-C. brms: An R Package for Bayesian Multilevel Models using Stan. Journal of Statistical Software., 2017,.

Ghosh, J., Li, Y., Mitra, R. On the Use of Cauchy Prior Distributions for Bayesian Logistic Regression. arXiv:1507.07170 [stat], 2017,.

Hilbe, J.M. Negative Binomial Regression. Cambridge University Press, Cambridge , 2011.

Kruschke, J.K. Doing Bayesian data analysis: a tutorial with R, JAGS, and Stan. Academic Press, Boston , 2015.

Kruschke, J.K. Rejecting or Accepting Parameter Values in Bayesian Estimation. Advances in Methods and Practices in Psychological Science, 2018, 1: 11.

Makowski, D., Ben-Shachar, M., Lüdecke, D. bayestestR: Describing Effects and their Uncertainty, Existence and Significance within the Bayesian Framework. JOSS, 2019, 4: 1541.

McCoy, B., Jahfari, S., Engels, G., Knapen, T., Theeuwes, J. Dopaminergic medication reduces striatal sensitivity to negative outcomes in Parkinson's disease. Brain, 2019, 142: 3605–3620.

Pirozzo, S. Whispered voice test for screening for hearing impairment in adults and children: systematic review. BMJ, 2003, 327: 967–0.

R Core Team R: A language and environment for statistical computing. R Foundation for Statistical Computing, Vienna, Austria , 2018.

Snellen, H. Probebuchstaben zur Bestimmung der Sehschärfe. Van de Weijer, Utrecht , 1862.

Stan Development Team Stan Modeling Language Users Guide and Reference Manual. , 2018.

Vehtari, A., Gabry, J., Magnusson, M., et al. loo: Efficient leave-one-out cross-validation and WAIC for Bayesian models. , 2020.

Vehtari, A., Gelman, A., Gabry, J. Practical Bayesian model evaluation using leave-one-out cross-validation and WAIC. Stat Comput, 2017, 27: 1413–1432.
